# Rationale for the Nodule Management Protocol of the Australian National Lung Cancer Screening Program

**DOI:** 10.1111/1754-9485.70044

**Published:** 2026-02-27

**Authors:** Mark W. McCusker, Samantha Ellis, Diane M. Pascoe, Catherine M. Jones, Stephen Melsom, Annette McWilliams, Tracy L. Leong, Henry Marshall, Kwun M. Fong, Fraser J. H. Brims, Miranda Siemienowicz

**Affiliations:** ^1^ Department of Radiology Royal Melbourne Hospital Melbourne Victoria Australia; ^2^ University of Melbourne Melbourne Victoria Australia; ^3^ Department of Radiology Alfred Health Melbourne Victoria Australia; ^4^ Department of Surgery Monash University Clayton Victoria Australia; ^5^ I‐Med Radiology Brisbane Queensland Australia; ^6^ Department of Medical Imaging Fiona Stanley Hospital Murdoch Western Australia Australia; ^7^ Perth Radiological Clinic Perth Western Australia Australia; ^8^ Department of Respiratory Medicine Fiona Stanley Hospital Murdoch Western Australia Australia; ^9^ Department of Respiratory Medicine Austin Health Heidelberg Victoria Australia; ^10^ Tumour Immunology Laboratory Olivia Newton‐John Cancer Research Institute Heidelberg Victoria Australia; ^11^ The Prince Charles Hospital University of Queensland Brisbane Queensland Australia; ^12^ Curtin Medical School Curtin University Perth Western Australia Australia; ^13^ National Centre for Asbestos Related Diseases Institute for Respiratory Health Perth Western Australia Australia; ^14^ Department of Respiratory Medicine Sir Charles Gairdner Hospital Nedlands Western Australia Australia; ^15^ Northern Imaging Victoria, Northern Health Epping Victoria Australia; ^16^ Central Clinical School Monash University Melbourne Victoria Australia

**Keywords:** chest CT, low‐dose CT, lung cancer, protocol, pulmonary nodules, screening

## Abstract

In July 2022, the Australian Medical Services Advisory Committee (MSAC) recommended the establishment of an Australian NLCSP (National Lung Cancer Screening Program), using low‐dose computed tomography (LDCT) for the early detection of lung cancer in asymptomatic high‐risk individuals. MSAC's recommendations for the NLCSP included core requirements as follows: (i) routine biennial screening, (ii) use of the PanCan risk prediction model for reporting baseline LDCT, and (iii) the use of the most recent version of Lung‐RADS for reporting follow‐up LDCT. Necessary adaptations were made to incorporate these requirements, resulting in a bespoke protocol for the Australian context. This article summarises the rationale and development of the NLCSP Nodule Management Protocol.

## 1 Background

In 2019, the Australian Minister for Health, the Hon Greg Hunt MP, invited Cancer Australia to conduct an enquiry into a potential National Lung Cancer Screening Program (NLCSP). On 29 July 2022, the Medical Services Advisory Committee (MSAC) recommended the establishment of an NLCSP, using low‐dose computed tomography (LDCT) for the early detection of lung cancer in asymptomatic high‐risk individuals [1, 2, 3, 4]. In 2023, the Australian Government announced funding of $263.8 million for the creation of the NLCSP.

The Cancer Australia application and MSAC recommendations for the NLCSP included the following core requirements: (i) routine biennial screening, (ii) use of the PanCan risk prediction model for reporting baseline LDCT, and (iii) the use of the most recent version of Lung‐RADS for reporting follow‐up LDCT. The rationale and supporting evidence for those requirements are detailed in the Cancer Australia Report on the Lung Cancer Screening Enquiry [1], the MSAC Public Summary Documents for National Lung Cancer Screening Program [2, 3], and in the Attachments to the MSAC Public Summary Document [4].

Cancer Australia contracted the Royal Australia and New Zealand College of Radiology (RANZCR) to synthesise a novel CT reporting protocol incorporating the above programme requirements, to guide radiologists reporting LDCT in the NLCSP. To perform this work, RANZCR was represented by the Australian and New Zealand Society of Thoracic Radiology (ANZSTR) a Special Interest Group within RANZCR. ANZSTR established a Lung Cancer Screening Working Group in the first quarter of 2024 which, in conjunction with representatives from the Thoracic Society of Australia and New Zealand (TSANZ) and the TSANZ Lung Cancer Working Group developed the NLCSP Nodule Management Protocol. The protocol is available at https://www.health.gov.au/resources/publications/nlcsp‐nodule‐management‐protocol.

This article summarises the rationale and development of baseline and follow‐up algorithms which comprise the NLCSP Nodule Management Protocol.

## 2 The Core Requirements of the NLCSP Nodule Management Protocol

### 2.1 Biennial LDCT for Participants With Very Low Risk Findings

The United States Preventive Services Task Force (USPSTF) and the American Cancer Society (ACS) recommend routine annual screening with LDCT [5, 6]. This approach is therefore incorporated in the Lung‐RADS categorisation and management system [7]. However, some of the more recently developed lung cancer screening programmes have employed routine biennial screening for selected low‐risk participants. These include the NHS Targeted Lung Health Checks Programme [8], and British Columbia Lung Screening Program [9].

The NELSON trial randomised 15,792 participants to either screening by LDCT, or no screening. For participants with a baseline screening LDCT demonstrating a largest nodule of less than 100 mm^3^, the probability of lung cancer after 2 years was very low, and similar to participants with no pulmonary nodules at baseline LDCT (0.6% vs. 0.4% respectively) [10]. This suggests that, for appropriately selected lung cancer screening participants, annual screening may not be necessary.

In addition, the NELSON investigators found that a 2‐year interval between the second and third screening rounds did not lead to a higher proportion of advanced stage lung cancers, compared to a 1‐year interval between the first and second screening rounds [11].

The MILD trial is the only randomised trial designed to compare the performance of different LDCT intervals for lung cancer screening [12, 13]. The study found that, in subjects with a negative baseline LDCT (83% of the MILD screening population), biennial lung cancer screening achieved similar outcomes to annual screening. Specifically, the mortality rate, proportion of stage II to stage IV cancers, and rate of interval cancers were similar between the annual and biennial screening arms [13]. In addition, 38% fewer LDCTs were performed in the biennial screening arm, compared to the annual screening arm [13].

Based on the above evidence suggesting similar clinical outcomes for appropriate participants, with biennial screening compared to annual screening, and their modelling of economic, clinical, and total cost outcomes, MSAC recommended biennial routine screening for the Australian NLCSP [2].

### 2.2 Use of the PanCan Lung Cancer Risk Prediction Model to Guide Baseline Management

The PanCan Lung Cancer Risk Prediction Model generates a probability, on a continuous scale, of a diagnosis of lung cancer within 2 and 4 years, using the following variables: age, sex, family history of lung cancer, presence of emphysema, nodule size, nodule morphology (ground glass, partially solid, or solid), upper lobe location, presence of spiculation, and total nodule count.

The parameters of the model were derived using logistic regression, from screening data from the Pan‐Canadian Early Detection of Lung Cancer Study [14]. The model has been validated in several studies, using data from chemoprevention trials of British Columbia Cancer [14], the Danish Lung Cancer Screening Trial [15], and the National Lung Cancer Screening Trial (NLST) [16].

The PanCan model is incorporated into some existing lung nodule management and lung cancer screening pathways, for example: British Thoracic Society guidelines [17], National Health Service England Targeted Lung Health Check Programme [8], American College of Radiology (ACR) Lung‐RADS v2022 [7], and the British Columbia Lung Cancer Screening Program [9].

The model does not include nodule growth or other temporal change in its calculation of malignancy risk. Therefore, if a screening participant has a prior CT scan available for comparison, the model should not be used. In the NLCSP, its use is reserved for interpretation of baseline LDCT only.

### 2.3 Use of ACR Lung‐RADS to Determine Follow‐Up Management

ACR Lung‐RADS [7] is a standardised reporting, management, and data collection system based on evidence and expert consensus, to guide lung cancer screening LDCT reporting. The first version was released in 2014. Updated versions released in 2019 and 2022 incorporated changes and additions, informed by accumulating evidence and experience [18, 19]. Lung‐RADS v2022 is the most recent iteration. Features of Lung‐RADS have been validated in several studies [20, 21, 22, 23, 24, 25].

Lung‐RADS includes six management categories (1, 2, 3, 4A, 4B, and 4X), assigned to individual pulmonary nodules based on size, morphology, and behaviour over time. A seventh category (Category 0) is assigned to studies that are incomplete or challenging to interpret due to suspected infection or inflammation. Increasing category number, from 1 to 4, reflects an increasing risk of cancer associated with that category. Each category is associated with a different management recommendation (see Table 1). The same categories and their associated management are used for reporting of both baseline and follow‐up LDCT. The screening participant's management is determined by the nodule with the highest risk Lung‐RADS category.

## 3 Management Categories and Terminology in the NLCSP

The NLCSP reporting algorithms incorporate the Lung‐RADS principle of consistent category numbering and associated management across baseline and follow‐up algorithms but introduce some changes in terminology and category numbering (see Table 1). These changes reflect the fact that, while based on Lung‐RADS v2022, the algorithms include several necessary deviations from Lung‐RADS v2022, to satisfy the MSAC requirements for the NLCSP. The category deviations adopted for the NLCSP include the introduction of an option for 24‐month follow‐up LDCT.

**TABLE 1:** Protocol categories and summarised management recommendations: Comparison between NLCSP and Lung‐RADS v2022.

NLCSP protocol			Lung‐RADS v2022		
NLCSP category	Category descriptor	Management	Lung‐RADS category	Category descriptor	Management
Category 0	Incomplete	1‐, 2‐, or 3‐month follow‐up for suspected infection or inflammation	Category 0	Incomplete	1‐to‐3‐month follow‐up for suspected infection or inflammation
Category 1	Very low risk	24‐month LDCT	Category 1	Negative	12‐month LDCT
Category 2	Low risk	12‐month LDCT	Category 2	Benign	12‐month LDCT
Category 3	Low to moderate risk	6‐month LDCT	Category3	Probably benign	6‐month LDCT
Category 4	Moderate risk	3‐month LDCT	Category 4A	Suspicious	3‐month LDCT
Category 5	High risk	Clinical referral	Category 4B	Very suspicious	Further investigations and/or clinical referral
Category 6	Very high risk	Clinical referral	Category 4X		Further investigations and/or clinical referral

## 4 Synthesis of the NLCSP Baseline LDCT Reporting Algorithm

### 4.1 Baseline Management of Nodules With Very Low, Low, and High PanCan Risk

The use of the PanCan model to define baseline categories and determine baseline management is a core requirement for the NLCSP [1, 2]. Specific strengths of the PanCan model are (i) the ability to define large groups of very low, and low‐risk screening participants, who can then undergo longer follow‐up intervals, and (ii) the ability to define a small group of high‐risk screening participants, who should be referred for clinical assessment [26, 27].

The International Lung Screening Trial (ILST) is the only prospective study to evaluate screening‐detected nodule management based on PanCan risk. Early data is displayed in Table 2. Of 4494 screening participants, a large majority (75% of participants) were assigned very low risk at baseline (PanCan risk < 1.5%) and were triaged to 24‐month follow‐up. Within 2 years of baseline, only eight lung cancers (0.2% of the group) were diagnosed. Conversely, a small group (3% of participants) were assigned high risk at baseline (PanCan risk ≥ 30% or findings deemed suspicious by the reporting radiologist) and were referred for clinical assessment. Within 2 years of baseline, 58 lung cancers (46% of the group) were diagnosed in the high‐risk group [26].

**TABLE 2:** Early data from the ILST: Lung cancers diagnosed within 2 years, for very low, low, and high‐risk baseline management categories [26].

Baseline management category	Pan Can risk	Baseline management	Number of participants (% of study cohort)	Number of cancers diagnosed within 2 years (% of category group)
Very low risk	< 1.5%	24‐month LDCT	3381 (75%)	8 (0.2%)
Low risk	1.5 to < 6%	12‐month LDCT	621 (14%)	10 (1.6%)
High risk	≥ 30% (or radiologist recommendation)	Clinical referral	125 (3%)	58 (46%)

The approach and findings of the ILST inform the baseline management for similarly defined groups in the NLCSP (see Table 3).

**TABLE 3:** NLCSP reporting recommendations for very low, low and high‐risk nodules at baseline.

Category descriptor	Baseline LDCT findings	NLCSP Category	Baseline management
Very low risk	Nodule with PanCan risk < 1.5%	1	24‐month LDCT
Low risk	Nodule with PanCan risk 1.5 to < 6%	2	12‐month LDCT
High risk	Nodule with PanCan risk ≥ 30%	5	Refer to Respiratory Physician linked to a lung cancer multidisciplinary team
Very high risk	Additional features suspicious for lung cancer	6	Refer to Respiratory Physician linked to a lung cancer multidisciplinary team

### 4.2 Baseline Management of Nodules With Intermediate PanCan Risk

The ILST defined a ‘moderate risk’ group of participants at baseline, who had nodules with PanCan risk of between 6% and < 30% [28]. The risk of cancer diagnosis within 2 years for this group was 8% [26]. As the risk for this group falls between the NLCSP low‐risk and high‐risk categories, it is reasonable to assign management between 12‐month LDCT and clinical referral. Therefore, the NLCSP retains the 6‐ and 3‐month LDCT management options used in Lung‐RADS.

An important consideration when employing shorter interval LDCT follow‐up durations is that some small lung cancers may not demonstrate measurable growth over short periods. Recent insights into the behaviour of small lung cancers over short follow‐up periods suggest that screen‐detected solid lung cancers with diameters measuring less than 6 mm on average may not demonstrate growth for up to two or more years [29]. For 76 screen‐detected lung cancers (median diameter 10 mm, range 5–34 mm; including solid and subsolid subtypes) only 7% demonstrated measurable growth at 3 months [30].

Lung‐RADS v2022 partially mitigates this problem by assigning follow‐up duration based on size [7]: solid nodules ≥ 6 to < 8 mm, and part solid nodules with ≥ 6 mm total mean diameter with solid component < 6 mm are assigned 6‐month follow‐up; solid nodules ≥ 8 to < 15 mm, and part solid nodules ≥ 6 mm total mean diameter with solid component ≥ 6 to < 8 mm are assigned 3‐month follow‐up [7]. Consequently, only larger nodules are followed up with a 3‐month LDCT, while smaller nodules, less likely to demonstrate growth at 3 months, are followed up with 6‐month LDCT.

The PanCan model determines risk based on multiple factors, not only nodule size and morphology. Therefore, it is possible that small nodules—including some solid nodules with diameters less than 6 mm—may generate a PanCan risk of between 6% and < 30%. Given they are unlikely to demonstrate measurable growth during that period, it is preferable to avoid 3‐month follow‐up LDCT for these small nodules.

The NLCSP baseline algorithm addresses this issue, by subdividing the group with PanCan risk 6% to < 30% risk group into two categories, one with PanCan risk of 6% to < 10%, and the other 10% to < 30%. This approach takes advantage of an important property of the PanCan model: nodules with a PanCan risk of 10% to < 30% are very unlikely to measure less than 8 mm.

The categories with PanCan risk of 6% to < 10% and 10% to < 30%, are assigned management of 6‐month LDCT and 3‐month LDCT respectively (see Table 4). Hence the baseline algorithm achieves a similar result as the Lung‐RADS v2022 size‐based cut‐offs and minimises sending smaller nodules for 3‐month follow‐up.

**TABLE 4:** NLCSP reporting recommendation for intermediate risk baseline nodules.

Category descriptor	Baseline LDCT findings	NLCSP category	Management
Low to moderate risk	Nodule with PanCan risk 6% to < 10%	3	6‐month LDCT
Moderate risk	Nodule with PanCan risk 10% to < 30%	4	3‐month LDCT

### 4.3 Baseline Management of Nodule Subtypes Not Accounted for by the PanCan Model

The PanCan model does not account for several specific nodule subtypes, where the morphology or location may confer a higher or lower risk of cancer. If applied to these nodules, the PanCan model may generate an inaccurate estimate of lung cancer risk. Therefore, these nodule subtypes are dealt with separately in the NLCSP Protocol. The subtypes are as follows: nodules with clearly benign features, juxtapleural nodules likely to be benign intrapulmonary lymph nodes, airway nodules, and atypical pulmonary cysts.

#### 4.3.1 Benign and Likely Benign Pulmonary Nodules

Clearly benign nodule features include benign calcification patterns and the presence of macroscopic fat. Complete, central, or concentric rim distribution is highly suggestive of benign granulomata, while the popcorn pattern of calcification or clear macroscopic fat content is highly suggestive of benign hamartomata [17].

Juxtapleural nodules are very likely to represent benign intrapulmonary lymph nodes if they demonstrate the following features: mean diameter < 10 mm or volume < 524 mm^3^; solid composition; smooth margins; oval, lentiform, or triangular shape; either contacting the pleura in any location, or located in the middle or lower lobes and within 15 mm of the pleura [31].

The NLCSP Protocol does not use the PanCan model for assessing benign and likely benign nodules. Instead, management in the baseline algorithm is follow‐up LDCT in 24 months (see Table 5).

**TABLE 5:** NLCSP reporting recommendations for baseline nodules with benign features.

Category descriptor	Baseline LDCT findings	NLCSP category	Baseline management
Very low risk	Nodule with benign features: Complete, central, popcorn or concentric ring calcifications Fat containing Juxtapleural nodule: 524 mm3 (< 10 mm) AND Solid; smooth margins; and oval, lentiform, or triangular shape	1	24‐month LDCT

#### 4.3.2 Airway Nodules

Airway nodules are rare in lung cancer screening populations, and the majority represent transient secretions that resolve on short interval follow‐up imaging [32]. However, airway cancers are easily overlooked by radiologists. In the NELSON trial, 22% of missed carcinomas began as airway lesions [33]. The NLCSP Protocol does not use the PanCan model for assessing airway nodules. Instead, the NLCSP Protocol incorporates specific baseline management guidance from Lung‐RADS v2022 for airway nodules (see Table 6) [7].

**TABLE 6:** NLCSP reporting recommendations for baseline airway nodules.

Category descriptor	Baseline LDCT findings	NLCSP category	Baseline management
Low risk	Airway nodule, subsegmental	2	12‐month LDCT
Moderate risk	Airway nodule, segmental or more proximal	4	3‐month LDCT

#### 4.3.3 Atypical Pulmonary Cysts

Primary lung cancers associated with cystic spaces are rare, and data on their natural history is limited [34]. Although awareness of this potential CT appearance of primary lung cancer is increasing, cystic lesions have posed a challenge to radiologists: 22% of missed cancers in the NELSON trial were initially associated with cystic spaces [33]. The NLCSP Protocol does not use the PanCan model for assessing atypical pulmonary cysts. Instead, the baseline management guidance is as per Lung‐RADS v2022 (see Table 7) [7].

**TABLE 7:** NLCSP reporting recommendations for baseline atypical pulmonary cysts.

Category descriptor	Baseline LDCT findings	NLCSP category	Baseline management
Moderate risk	Atypical pulmonary cyst: Thick‐walled cyst or Multilocular cyst	4	3‐month LDCT

## 5 Synthesis of the NLCSP Follow‐Up LDCT Reporting Algorithm

As recommended by MSAC, the follow‐up algorithm for the NLCSP is based on Lung‐RADS v2022. The algorithm includes necessary adaptations to accommodate the requirement for very low‐risk findings to undergo routine screening every 2 years.

### 5.1 Changes to Stepped Management for Stable Nodules

The NLCSP follow‐up algorithm incorporates the stepped management approach from Lung‐RADS v2022, whereby stable nodules are reassigned a lower category and progressively lengthier follow‐up periods.

For stable NLCSP Category 3 and 4 nodules, the process is identical for stable Lung‐RADS v2022 Category 3 and 4 nodules [18] (see Table 8). Note that the stepped management approach does not apply to persistent segmental or proximal airway nodules, as these are considered high risk even when stable.

**TABLE 8:** Stepped approach to the management of stable NLCSP Category 3 and 4 nodules. Note that the stepped approach does not apply to airway nodules.

Category descriptor	Follow‐up LDCT findings	NLCSP category	Follow‐up management
Low risk	Previous Category 3 lesion that is stable or decreased in size at 6‐month follow‐up LDCT	2	12‐month LDCT
Low to moderate risk	Previous Category 4 lesion (excluding persistent segmental or proximal airway nodules) that is stable or decreased in size at 3‐month follow‐up LDCT	3	6‐month LDCT

For eligible stable nodules to return to biennial screening via the stepped approach, modifications to the Lung‐RADS v2022 Category 2 management are necessary. This is because biennial screening should be reserved only for those participants with a very low risk of cancer within 2 years. For higher risk participants, a prolonged screening interval is less effective and potentially results in more interval cancers and late‐stage screen‐detected cancers [35].

At baseline, the PanCan Calculator is very effective at selecting very low risk participants for 24‐month LDCT. A similar risk threshold is required at follow‐up, to send participants to 24‐month LDCT. In the absence of evidence to support any specific approach, the authors reason that for a stable nodule to return to NLCSP Category 1, and be followed with 24‐month LDCT, it should demonstrate stability for a period of at least 24 months. This approach will be reviewed as the NLCSP matures.

This rationale results in the requirement for an NLCSP Category 2 lesion, stable for less than 24 months, to remain Category 2, and receive an additional 12‐month follow‐up LDCT (see Table 9).

**TABLE 9:** Modified approach to the management of stable NLCSP Category 1 and Category 2 lesions.

Category descriptor	Follow‐up LDCT findings	NLCSP category	Follow‐up management
Very low risk	Previous Category 2 lesion that is stable or decreased in size *over a period of 24 months or more*	1	24‐month LDCT
Low risk	Previous Category 2 lesion that is stable or decreased in size *over a period of less than 24 months*	2	12‐month LDCT

### 5.2 Growth Definitions

Lung nodule enlargement on CT, determined by a measured increase in either volume or diameter, is an important predictor of lung cancer [36, 37]. Lung‐RADS v2022 defines ‘growth’ as an increase in mean diameter size of > 1.5 mm (or increase in volume > 2 mm^3^) within a 12‐month interval. Lung‐RADS v2022 also introduced an additional definition, for ‘slow growth’ to detect and trigger referral for indolent lung cancers. ‘Slow growth’ is defined as enlargement over multiple screening studies but not meeting the > 1.5 mm threshold increase within 12 months [7].

To apply the provisions of Lung‐RADS v2022 for follow up, the NLCSP Protocol preserves the distinction between ‘growth’ and ‘slow growth’. However, the exact Lung‐RADS v2022 definitions are not suitable for the NLCSP for the following reasons:

1. In the NLCSP the maximum potential follow‐up interval is 24 months, in contrast to 12 months in Lung‐RADS v2022. The Lung‐RADS v2022 definition of growth requires enlargement within a 12‐month period but causes ambiguity when applied to nodules that enlarge over a 12‐ to 24‐month period. For example, if a nodule increases by 1.7 mm over 24 months, it is not clear if this represents ‘growth’ or ‘slow growth’ by the Lung‐RADS v2022 definition.
2. Annual screening in Lung‐RADS v2022 permits more frequent opportunities to discover slow growing nodules, and therefore allows a more specific definition for growth, as every nodule defined as stable nodule will still be assessed at least every 12 months. The NLCSP use of a 24‐month follow‐up period means that a nodule defined as stable might not be imaged again for another 2 years, so it is prudent to introduce a higher threshold for the definition of ‘stability’.

#### 5.2.1 NLCSP Definitions Based on Nodule Volumetry

##### 5.2.1.1 Threshold for True Growth by Relative Change in Volume

Multiple factors influence the measurement of pulmonary nodule volume on CT, including CT scanner model, image acquisition and reconstruction parameters, software used for volumetry, nodule position and characteristics, patient factors, and observer training and experience [38]. Any of these factors can introduce variability in nodule volume measurement. To determine reliably if interval growth has occurred, studies and programmes using nodule volumetry have set thresholds for percentage volume change required to demonstrate true change [39]. The most commonly used volume range of variability is a relative increase or decrease of 25% or more [40, 41, 42]. This threshold is employed in the NELSON trial [43], UK SUMMIT Trial [44], BTS guidelines [17], NHS Targeted Lung Health Checks Programme [8].

A study examining variability in volume measurement of small non‐metastatic pulmonary nodules has questioned the 25% threshold and suggested that a volume increase of only 15% may indicate true growth. However, all participants in the study were imaged on the same CT machine, and their images were analysed by the same radiologists using the same segmentation software [45]. The results are not generalisable to the NLCSP context, which will involve more variability.

As such, the NLCSP uses the 25% volume change cut‐off to determine true change. An increase of 25% or more represents ‘growth’ or ‘slow growth’, while a reduction of 25% or more is considered ‘decrease’ (see Table 10).

**TABLE 10:** Definitions of ‘growing’, ‘slowly growing’, ‘stable’, and ‘decreased’ in the NLCSP follow‐up algorithm.

	Nodule measurement using volumetry (preferred method)	Nodule measurement using mean diameter (when measurement using volumetry is not possible)
‘Growing’	Increase greater than 25% *and* VDT < 600 days	Increase of more than 1.5 mm, over 24 months or less
‘Slowly growing’	Increase greater than 25% *and* VDT > 600 days on *more than one scan interval*	Increase of more than 1.5 mm, over *more* than 24 months
‘Stable’	Change between −25% and +25% *or* increase from baseline greater than 25% and VDT > 600 days but not slow growth	Change of between −1.5 mm and +1.5 mm
‘Decreased’	Decrease of 25% or more	Decrease of 1.5 mm or more

##### 5.2.1.2 Volume Doubling Time to Distinguish ‘Growth’ and ‘Slow Growth’

Volume doubling time (VDT) is an important parameter for predicting malignancy in lung nodules and differentiating slow‐growing cancers from more rapidly growing cancers. VDT measurement has been used in the management protocols of several lung cancer screening studies [46, 47, 48, 49, 50] and incorporated into the protocol of the NHS Targeted Lung Health Checks Programme [8].

The NELSON trial used VDT to stratify risk for enlarging nodules with a volume between 100 and 300 mm^3^. Those with a VDT of 400 days or fewer, VDT of 400 to 600 days, and VDT of greater than 600 days had cancer probabilities of 9.9%, 4%, and 0.8% respectively [10]. A VDT cut‐off of 400 days is frequently referenced as the upper limit to distinguish benign from malignant nodules [39].

However, there is evidence that VDT varies with nodule CT features and lung cancer subtype [37, 51]. Screen‐detected lung cancers with a subsolid morphology have significantly longer VDTs compared to those with solid morphology [37]. Screen‐detected adenocarcinomas have been shown to exhibit longer VDTs than small cell and squamous cell lung cancers [37], and there is evidence that some subtypes of adenocarcinoma (lepidic, acinar, and papillary) have much longer VDTs than others (solid and micropapillary) [51]. In addition, there are many reports of subgroups of lung cancer with VDTs much longer than 400 days [37, 51, 52].

In Lung‐RADS v2022, and in the NLCSP follow‐up algorithm, ‘growth’ is a critical variable to prompt clinical referral, while ‘stability’ will result in the stepped approach of lengthening follow‐up scan intervals. For a biennial programme, it is important that only the lowest risk nodules are managed with 24‐month follow‐up. To limit sending very slowly enlarging lung cancers to biennial follow‐up, the use of a more sensitive, less specific definition of growth is prudent.

In the absence of evidence to support a specific volume‐based growth definition for biennial lung cancer screening, the definition of ‘growing’ selected for the NLCSP is ‘volume increase greater than 25% *and* VDT less than 600 days’ (see Table 10). This sacrifices some of the specificity of the 400‐day threshold but achieves an increase in sensitivity. This definition will avoid sending some slow‐growing lung cancers for 24‐month follow‐up.

The NLCSP, like Lung‐RADS v2022, incorporates a distinct definition for ‘slow growing’ and the Lung‐RADS v2022 requirement for change over two scan intervals, but bases the definition on VDT as follows: ‘a volume increase of greater than 25% *and* VDT greater than 600 days on *more than one scan interval*’ (see Table 10).

#### 5.2.2 NLCSP Definitions Based on Measurement of Mean Nodule Diameter

Nodule measurement using volumetry is the preferred approach in the NLCSP. But there will be circumstances under which the use of volumetry is not possible, for example if contouring cannot be applied accurately (for some low‐density lesions, or some lesions abutting a vessel or pleura), or if it is not available. In these circumstances, nodules should be measured manually by calliper, in both the long and short axis, and reported as mean nodule diameter. The orthogonal plane (axial, sagittal, or coronal) used for manual measurement should be the one that most accurately reflects the nodule shape and size.

The Lung‐RADS v2022 definitions for ‘growth’ and ‘slow growth’ have been adapted as follows: ‘growing’ is defined as an increase in mean diameter of more than 1.5 mm, over 24 months or less, while ‘slowly growing’ is defined as an increase in mean diameter of more than 1.5 mm, over *more* than 24 months (see Table 10).

## 6 Further Investigation for High Risk and Very High‐Risk Categories

The management in Lung‐RADS v2022 for categories 4A, 4B, and 4X includes options for PET CT and tissue sampling. In the NLCSP, screening activity ends at the time of clinical referral. Any further investigations are determined by the appropriate specialist or multidisciplinary team and are not considered screening. Hence, the NLCSP protocol does not include any further management or investigations beyond specialist referral.

## 7 Explanatory Notes for the Protocol

Detailed Explanatory Notes accompany the baseline and follow‐up algorithms of the NLCSP Nodule Management Protocol. These are based on similar notes in Lung‐RADS v2022 but contain relevant amendments and additions for the NLCSP context. The notes include definitions of specific terms used in the protocol, guidance on the approach to nodule measurement, suggestions on the approach to using historical imaging, and guidance for situations when scans are missed or delayed.

## 8 Evaluation of the Protocol

Following the launch of the NLCSP on 1 July 2025, the Nodule Management Protocol will be assessed in a real‐world setting. A comprehensive set of Performance Indicators is being developed by the Department of Health, Disability and Ageing, leveraging standardised data submitted to the National Cancer Screening Register using the NLCSP Structured Clinical Radiology Report for participants in the programme. Cancer Australia has established a research consortium that will specifically examine the clinical performance of the Protocol, while the Commonwealth Government has committed to evaluate the entire programme (including the Protocol) after 2 years.

## 9 Conclusion

The Australian NLCSP Nodule Management Protocol incorporates the MSAC requirements for biennial screening, use of the PanCan Lung Cancer Risk Prediction Model for baseline management, and the use of Lung‐RADS v2022 for follow‐up management. Necessary adaptations have been made to incorporate these requirements. The result is a bespoke nodule management protocol for the Australian context. As the NLCSP develops, increasing experience and evidence will be used to inform future changes.

## Author Contributions

**Mark W. McCusker:** conceptualization; methodology; writing – original draft; writing – review and editing; visualization. **Samantha Ellis:** conceptualization; methodology; visualization; writing – review and editing. **Diane M. Pascoe:** writing – review and editing; conceptualization; methodology; visualization. **Catherine M. Jones:** conceptualization; methodology; visualization; writing – review and editing. **Stephen Melsom:** conceptualization; methodology; visualization; writing – review and editing. **Annette McWilliams:** writing – review and editing; conceptualization; methodology. **Tracy L. Leong:** writing – review and editing; conceptualization; methodology. **Henry Marshall:** writing – review and editing. **Kwun M. Fong:** writing – review and editing. **Fraser J. H. Brims:** writing – review and editing. **Miranda Siemienowicz:** conceptualization; methodology; visualization; writing – review and editing.

## Conflicts of Interest

The authors declare no conflicts of interest.

## Data Availability

Data sharing not applicable to this article as no datasets were generated or analysed during the current study.
